# ^18^F-MK-9470 PET imaging of the type 1 cannabinoid receptor in prostate carcinoma: a pilot study

**DOI:** 10.1186/2191-219X-3-59

**Published:** 2013-08-02

**Authors:** Kimy M Emonds, Michel Koole, Cindy Casteels, Laura Van den Bergh, Guy M Bormans, Filip Claus, Liesbeth De Wever, Evelyne Lerut, Hendrik Van Poppel, Steven Joniau, Herlinde Dumez, Karin Haustermans, Luc Mortelmans, Karolien Goffin, Koen Van Laere, Christophe M Deroose, Felix M Mottaghy

**Affiliations:** 1 Department of Nuclear Medicine, University Hospitals Leuven, Leuven 3000, Belgium; 2 Department of Radiotherapy, University Hospitals Leuven, Leuven 3000, Belgium; 3 Laboratory for Radiopharmacy, KU Leuven, Leuven 3000, Belgium; 4 Department of Radiology, University Hospitals Leuven, Leuven 3000, Belgium; 5 Department of Morphology and Molecular Pathology, University Hospitals Leuven, Leuven 3000, Belgium; 6 Department of Urology, University Hospitals Leuven, Leuven 3000, Belgium; 7 Department of Medical Oncology, University Hospitals Leuven, Leuven 3000, Belgium; 8 Department of Nuclear Medicine, University Hospital of Aachen, Aachen 52074, Germany; 9 Department of Nuclear Medicine, Maastricht University Medical Center, Maastricht 6200, the Netherlands

**Keywords:** Prostate cancer, CB1R, PET/CT, MRI, ^18^F-MK-9470

## Abstract

**Background:**

Preclinical and histological data show overexpression of the type 1 cannabinoid receptor (CB1R) in prostate carcinoma (PCa). In a prospective study, the feasibility of ^18^F-MK-9470 positron emission tomography (PET) imaging in patients with primary and metastatic PCa was evaluated.

**Methods:**

Eight patients were included and underwent ^18^F-MK-9470 PET/CT imaging. For five patients with primary PCa, dynamic PET/CT imaging was performed over three acquisition intervals (0 to 30, 60 to 90 and 120 to 150 min post-injection). In malignant and benign prostate tissue regions, time activity curves of the mean standardized uptake value (SUV_mean_) were determined as well as the corresponding area under the curve to compare ^18^F-MK-9470 uptake over time. Muscle uptake of ^18^F-MK-9470 was used as reference for non-specific binding. Magnetic resonance imaging (MRI) was used as anatomical reference and for delineating intraprostatic tumours. Histological and immunohistochemical (IHC) examination was performed on the whole-mount histopathology sections of four patients who underwent radical prostatectomy to assess the MRI-based tumour versus benign tissue classification. For three patients with proven advanced metastatic disease, two static PET/CTs were performed 1 and 3 h post-injection. ^18^F-MK-9470 uptake was evaluated in bone lesions of metastatic PCa by comparing SUV_mean_ values of metastases with these of the contralateral bone tissue.

**Results:**

^18^F-MK-9470 uptake was significantly higher in benign and malignant prostate tissue compared to muscle, but it did not differ between both prostate tissue compartments. IHC findings of corresponding prostatic histopathological sections indicated weak CB1R expression in locally confined PCa, which was not visualized with ^18^F-MK-9470 PET. Metastases in the axial skeleton could not be detected while some metastases in the appendicular skeleton showed higher ^18^F-MK-9470 uptake as compared to the uptake in contralateral normal bone.

**Conclusions:**

^18^F-MK-9470 PET could not detect local PCa or bone metastases in the axial skeleton but was able to visualize metastases in the appendicular skeleton. Based on these pilot observations, it seems unlikely that CB1R PET will play a significant role in the evaluation of PCa.

## Background

Prostate carcinoma (PCa) is the second most frequently diagnosed cancer and ranks as the sixth leading cause of cancer death in men worldwide [1]. To date, treatments including radical prostatectomy (RP) and radiotherapy (RT) intend to cure localized PCa; however, within 2 to 4 years recurrent, mostly incurable disease occurs in 30% to 50% of all patients. Investigating the contribution of new molecular targets at various stages of the disease as novel diagnostic and therapeutic approaches is warranted.

The past years, the endocannabinoid system (ECS) has been one of the targets of interest in the field of oncology due to the discovery of diverse ECS-mediated pathways in tumour cells including a role in the stimulation of apoptosis and inhibition of tumour growth, invasion and migration [2-8]. Endocannabinoid effects occur upon the activation of mainly two specific G protein-coupled receptors - the type 1 cannabinoid (CB1R) and type 2 cannabinoid (CB2R) receptor. Whereas CB1R is ubiquitous in the brain, it is also widely expressed peripherally. Under normal conditions, CB2R is almost exclusively located outside the brain in lymphoid tissues [3]. Although an elevated expression of both cannabinoid receptors has been demonstrated *in vitro* in PCa cell lines compared to the expression levels in normal prostatic cells [3], most research efforts to date have concentrated on the clinical potential of CB1R in PCa. CB1R expression does not correlate with increasing tumour grade [9]. Yet, increased CB1R levels were demonstrated in human PCa specimens compared to those of the benign samples [10]. Also, high CB1R immunoreactivity was associated with a higher incidence of metastasis and high Gleason score at initial diagnosis and with a lower disease-specific survival [10]. Increased CB1R density was further related to increased levels of fatty acid amide hydrolase and phosphorylated epidermal growth factor receptor (pEGFR) expression in PCa specimens. The potential of CB1R-based therapeutics reducing cannabinoid metabolism and tumour proliferation was discussed [11,12].

In view of the recent development of several radioligands for CB1R imaging that were mainly investigated in the central nervous system [13-19], we set up a prospective study to detect CB1R in patients with PCa using the radioligand ^18^F-MK-9470. ^18^F-MK-9470 has high selectivity and affinity for CB1R (Ki = 0.9 nM). Also, this radioligand shows mainly a hepatobiliary excretion [20] with only a small fraction of renal excretion, which might be advantageous for PET imaging of orthotopic prostate tissue (benign and malignant). Whole body ^18^F-MK-9470 PET imaging did not show specific bone marrow and skeletal uptake. Therefore, we assume that this agent could be useful in the detection and molecular characterization of PCa skeletal metastases [20].

The present study aimed to evaluate the utility of ^18^F-MK-9470 PET/CT to detect CB1R expression in both primary and metastatic PCa. Based on previous observations in clinical specimen, the hypothesis was that CB1R expression would be increased in locally confined PCa compared to benign prostate tissue, and that this would result in increased ^18^F-MK-9470 uptake in primary lesions. Furthermore, we wanted to evaluate the potential of ^18^F-MK-9470 PET/CT to detect PCa bone metastases.

## Methods

### Patients

Eight patients, with a Karnofsky score of at least 80, were recruited. Five of these patients had biopsy-proven PCa and had no previous treatment. Prostate tumours were at least stage T2b and had a diameter ≥ 1.4 cm, as defined by sonography or magnetic resonance imaging (MRI). Four out of these five patients had locally confined PCa and were scheduled for RP while one patient presented with a solitary bone metastasis on the bone scan. Three patients with disease recurrence after primary therapy, a prostate specific antigen (PSA) level of more than 3 ng/mL and a positive bone scan were further included. All patients gave written informed consent, and the study was approved by the local ethics committee.

### Imaging

#### Radiochemical synthesis of ^18^F-MK-9470

^18^F-MK-9470, (N-[2-(3-cyano-phenyl)-3-(4-(2-[^18^F]fluoroethoxy)phenyl)-1-methylpropyl]-2-(5-methyl-2-pyridyloxy)-2-methylproponamide) [21], is an inverse agonist which has shown a high selectivity and specificity for the human CB1R [14]. The precursor for tracer synthesis was obtained from Merck Research Laboratories (MRL, West Point, PA, USA), and radiolabelling was done by alkylation with 2-[^18^F]fluoroethylbromide [21]. After high-performance liquid chromatography (HPLC) separation, an end product with a radiochemical purity of > 95% and a specific activity of > 20 GBq/μmol was produced.

#### ^18^F-MK-9470 metabolite analysis

To evaluate peripheral metabolites, venous samples were taken at 5, 10, 20, 40 and 60 min after ^18^F-MK-9470 injection. Acetonitrile (1.2 ml) was added to 1 ml plasma. Next, the mixture was centrifugated to precipitate protein content whereafter 1.5 ml of the supernatant was filtered (Millipore GV 0.22 μm, Billerica, MA, USA, 13 mm diameter). A solution consisting of 1 mL filtered supernatant and 20 μl cold product was injected onto the HPLC system (Waters C18 XTerra, Milford, MA, USA, 5 μm, 4.6 × 250 mm, 1.5 ml/min, 50:50 acetonitrile/50 mM sodium acetate, pH 5.5). HPLC eluants from 0 to 6.5 min (fraction A, metabolite fraction) and 6.5 to 12 min (fraction B, parent fraction) were collected. The amount of radioactivity in both fractions was counted in a gamma counter (Wallac Wizard 3″ 1480, PerkinElmer, Turku, Finland).

#### PET/CT imaging

PET/CT studies were performed using a Siemens Biograph Hirez 16-slice LSO PET-CT system (Siemens Medical, Erlangen, Germany). Patients fasted for at least 4 h before ^18^F-MK-9470 administration. An intravenous bolus injection of ^18^F-MK-9470 (mean ± SD: 289 ± 22 MBq) was performed through an antecubital vein.

For five patients with primary PCa, a 30-min dynamic PET/CT acquisition over the pelvis was performed starting at the time of injection (first interval), at 60 (second interval) and 120 (third interval) min after the administration of ^18^F-MK-9470. One low-dose and two ‘minimal’ dose CT scans (slice separation of 3 mm, slice thickness of 5 mm; 30 versus 9 mAs) were performed preceding each PET acquisition. Patients with metastatic PCa (*n* = 3) received two static whole-body PET scans (six bed positions, 5 min scanning time per position), starting at 60 and 180 min post-injection (p.i.). These patients received a contrast-enhanced whole-body CT scan (slice separation of 3 mm, slice thickness of 5 mm) for delineation of metastatic tissue at 60 min and a ‘minimal’ dose CT (slice separation of 3 mm, slice thickness of 5 mm; 9 mAs) at 180 min for attenuation correction only. The contrast-enhanced whole-body CT scan was performed with 120 ml non-ionic contrast agent administered intravenously as a bolus (Ultravist, Schering, Berlin, Germany: injection rate of 1.6 ml/s followed by 35 ml NaCl 0.9% in 100 s).

PET images were iteratively reconstructed with ordered subsets expectation maximization (five iterations, eight subsets) with a three-dimensional (3D) Gaussian post-reconstruction smoothing of 5 mm. CT-based attenuation and scatter correction was performed.

### Magnetic resonance imaging

MRI was performed using a 1.5T MR unit (Sonata Vision; Siemens, Erlangen, Germany) with a combination of a six-channel phased array body coil and spine coil. All MRI data sets were extended from the promontorium to the penile bulb. T_2_-weighted (T_2_w) turbo spin echo images were obtained in the transverse, coronal and sagittal plane as an anatomical reference (scan parameters: repetition time/echo time = 6,000/136 ms, slice thickness of 3 mm, voxel size = 0.7 × 0.6 × 3.0 mm, 5 averages, acquisition time approximately 6 min). For primary tumour detection and delineation, an echo-planar diffusion-weighted imaging (DWI) sequence was used in a transverse plane with following parameters: 4 mm slice thickness, voxel size 3.0 × 3.0 × 4.0 mm, acquisition time approximately 5 min. Six *b* values were applied (*b* = 0, 50, 100, 500, 750 and 1,000 s/mm^2^) to calculate the apparent diffusion coefficient (ADC). On this ADC map, a threshold of 95 × 10^−5^ mm^2^/s was used to discriminate tumoural tissue from non-malignant prostatic tissue.

### Pathological examinations and immunohistochemistry

Tissue sections obtained after RP were examined microscopically after routine preparation [22-26]. Immediately after surgical removal, the RP specimen was brought to the pathology lab. Following fixation in 6% formalin (24 h, room temperature), tissues were examined macroscopically. The prostate was inked to allow proper orientation, weighted and sectioned at 3-mm intervals perpendicular to the urethra allowing comparison with image slices. Tissue sections were embedded into macrocassettes for further processing. One cross section from each processed tissue section was examined on hematoxylin-eosin stain. All tumour regions were outlined on the glass slide by an experienced uropathologist (EL). The Gleason score was assigned [27], and pathological tumour stage was determined according to the TNM 2009 classification.

For IHC, antigen retrieval in the paraffin-embedded sections (5 μ) was done automatically (Dako PT Link, Dako, Denmark). Sections were subsequently rinsed in phosphate buffered saline (PBS). After removal of endogenous peroxidase (Flex Dako, Ready to Use, Dako) and rinsing in PBS, sections were incubated with polyclonal anti-CB1R receptor antibody (Abcam cat no. 23703, dilution 1:100, 30 min at room temperature), again rinsed in PBS, and then incubated with the secondary system (Dual-Linked Envision (Dako) and benzedine (Envision Flex, Dako), 30 min at room temperature).

Assessment of the immunohistochemical stains was done semiquantitatively by an experienced uropathologist (EL): negative, weak, moderate and strong positivity was noted both in the tumour as in the surrounding non-tumoural prostatic epithelium. Furthermore, it was noted which cell compartment expressed CB1R (nucleus, cytoplasm, cell membrane) and whether CB1R positivity was homogeneously distributed within the prostate.

### Image fusion and data analysis

Analysis of all imaging data was done with PMOD 3.1 software (PMOD Inc., Zurich, Switzerland).

#### Primary PCa

**Figure 1:**
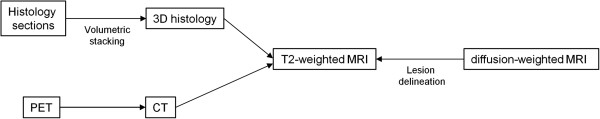
Schematic illustration of 3D image coregistrations performed in this study.

Figure 1 schematically demonstrates the various 3D coregistration procedures of the different image modalities performed in this study.

MRI images were used as anatomical reference to visualize the prostate and differentiate PCa and normal prostate tissue (volumes of interest (VOI)). For accurate delineation of the prostate, the CT scan of the PET/CT was coregistered automatically with the T_2_w MRI scan of the pelvis using a mutual information algorithm. VOIs for PCa and benign prostatic tissue were delineated on the transaxial slices of DWI images and then transferred onto the corresponding matched T_2_w MRI data set. To coregister ^18^F-MK-9470 PET with MRI, the transferred VOIs on the T_2_w MRI data set were positioned onto the coregistered PET/CT data set in order to quantify ^18^F-MK-9470 uptake in PCa and benign prostatic tissue. The mean standardized uptake value (SUV_mean_) in these VOIs was determined. The uptake of ^18^F-MK-9470 in muscle tissue was used as reference for non-specific binding to calculate the relative uptake in PCa and benign prostatic tissue, as the CB1R is not expressed in muscle [28]. Time activity curves of SUV_mean_ values were determined in order to calculate the area under the curve (AUC) for each VOI representing a specific region (PCa, benign and muscle).

Histological cross sections, corrected for fixation shrinkage (shrinkage factor 1.33), were merged (3D volumetric stacking); after which, the 3D histology image was visually reoriented perpendicular to the urethra on the T_2_w MRI image. Subsequently, sections of the 3D histology image were reoriented manually to align with the anatomical slice of the prostate on the T_2_w MRI (that was previously coregistered to the ^18^F-MK-9470 PET data set).

#### Metastatic PCa

Tumour and contralateral normal bone tissue was delineated on the coronal plane of PET/CT images of patients with metastatic PCa, using a previously obtained bone scan as reference for tumour localization. The SUV_mean_ was calculated in the specific bone volumes.

Delineation of a metastasis detected at initial diagnosis was done based on the T_2_w MRI image as it was located in the field of view (left femoral head). Based on the SUV_mean_ values, the AUC of ^18^F-MK-9470 uptake in malignant and contralateral normal bone was calculated.

### Statistical analysis

Statistical analysis was performed using Graphpad Prism (San Diego, CA, USA). To compare the uptake of ^18^F-MK-9470 in locally confined PCa, benign prostatic tissue and muscle by means of the AUC values of each time interval, the paired one-way ANOVA and Bonferroni post hoc testing were used. ^18^F-MK-9470 uptake (mean ± standard error of the mean (SEM)) in lesions of the axial skeleton was compared with the uptake in normal axial bone tissue using the Student’s *t* test. Comparison of PCa volumes (mean ± SEM) delineated on the ADC MRI and on histology was done using the paired two-sided Student’s *t* test. *P* values ≤ 0.05 indicate significance.

## Results

Patient characteristics are summarized in Tables 1 and 2. The median patient age was 62.5 years (range 57 to 73 years). The median serum PSA of patients with initial diagnosis of PCa was 4 ng/mL (range 1.3 to 8 ng/mL).

**Table 1:** Patient characteristics in primary PCa

**ID**	**Age (years)**	**Primary PCa**		
		**TNM**	**GS**	**Preoperative PSA (ng/ml)**
1	63	pT2cN0	7 (4 + 3)	7.4
2	65	pT3aN0	8 (4 + 4)	1.3
3	62	pT3aN0	8 (4 + 4)	4
4	57	pT2cN1	7 (4 + 3)	8
5	57	cT4N1M1	9 (5 + 4)	3.32^a^

^a^Bone metastasis at left femoral head. PSA determined at initial diagnosis. *GS*, Gleason score.

**Table 2:** Patient characteristics in metastatic PCa

**ID**	**Age (years)**	**Metastatic PCa**	
		**Primary Tx**	**BM**
6	58	AD	Axial skeleton, humeri, femora
7	73	RT	Axial skeleton, sternum, left acromioclavicular and right sternoclavicular joint
8	64	AD	Axial skeleton, scapula, left proximal femur diaphysis

Tx, treatment; BM, bone metastasis.

### Primary PCa

**Figure 2:**
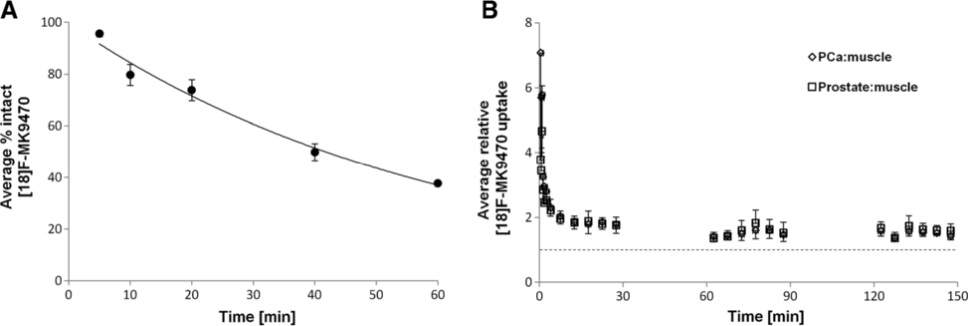
^**18**^**F-MK-9470 metabolism and relative kinetics in prostate tissue.** Average fraction intact ^18^F-MK-9470 in venous samples of five patients **(A)**. Relative ^18^F-MK-9470 kinetics in tumour and benign prostatic tissue compared to muscle at three acquisition intervals (0 to 30, 60 to 90 and 120 to 150 min p.i.) **(B)**.

^18^F-MK-9470 metabolism in venous samples of patients 1 to 5 is demonstrated in Figure 2A. At 5 min p.i., 96% ± 1% of the total radioactivity in venous plasma corresponded to intact ^18^F-MK-9470. This fraction declined to 80% ± 4% at 10 min p.i., 74% ± 4% at 20 min p.i., 50% ± 3% at 40 min p.i. and 38% ± 1% at 60 min p.i.

**Figure 3:**
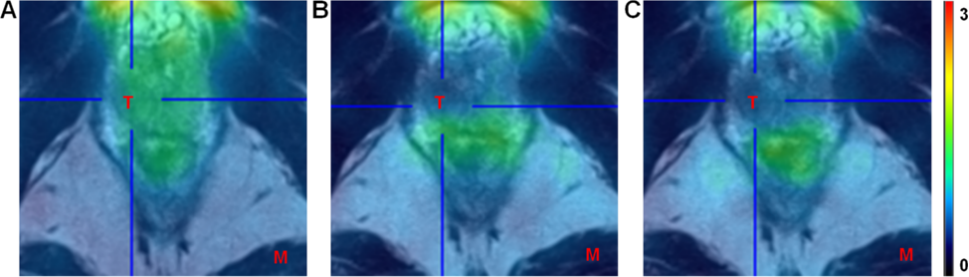
**PET/MRI imaging in locally confined PCa.** PET/MRI fusion image of the first **(A)**, second **(B)** and third **(C)** acquisition time interval in a representative patient (patient 4). The uptake of ^18^F-MK-9470 in the local tumour is comparable to the uptake in the surrounding healthy prostatic tissue. T, tumour; M, muscle. Data are scaled from 0 to 3 SUV.

The uptake of ^18^F-MK-9470 over time in tumour and benign prostatic regions compared to the uptake in muscle tissue is demonstrated in Figure 2B. Based on the AUC, no significantly different ^18^F-MK-9470 uptake was observed between tumoural and benign prostatic regions; nevertheless, the uptake in both prostate compartments was significantly higher than that in the muscle tissue in the first (0 to 30 min, *p* < 0.05) and last (120 to 150 min, *p* < 0.05) acquisition time interval. Table 3 presents the mean AUC values of each specific region (PCa, benign prostate, muscle) over time. Figure 3 illustrates the comparable uptake of ^18^F-MK-9470 in the local tumour as compared with the uptake in the surrounding healthy prostatic tissue.

**Table 3:** **Mean area under the curve values (SUV**_**mean **_**× min)**

	**PCa**	**Benign**	**Muscle**
0 to 30 min	30.9 ± 3.1	29.9 ± 3.0	16.0 ± 1.9
60 to 90 min	18.5 ± 0.9	19.1 ± 2.5	12.8 ± 1.0
120 to 150 min	17.5 ± 0.6	18.3 ± 1.8	11.8 ± 0.5

**Figure 4:**
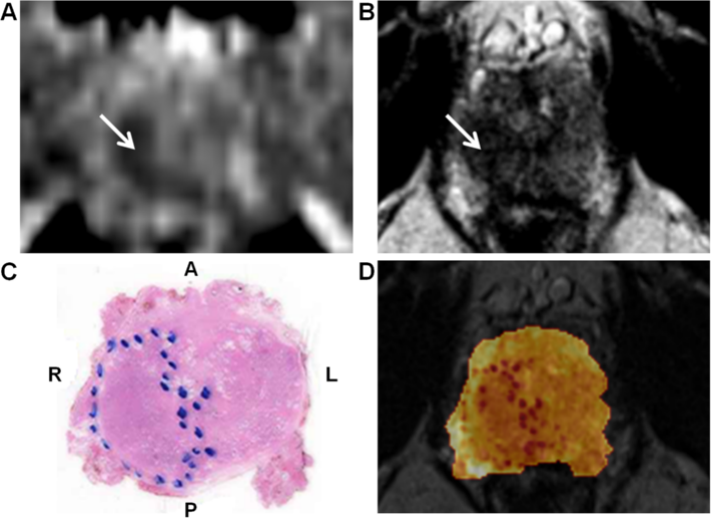
**Coregistration of anatomical imaging and histology.** Prostatic MRI examination in patient 4. ADC map shows restricted diffusion in low-signal tumour mass in the right peripheral zone (arrow) **(A)**. The corresponding transversal T_2_w MRI image shows a low-signal-intensity mass (arrow) **(B)**. Photomicrograph from a tissue cross section obtained after RP. The tumoural lesion on the right is outlined (blue dots) **(C)***.* Fusion of the transverse T_2_w MRI image with the corresponding tissue cross section **(D)**.

PCa localized on the DWI MRI images was visually confirmed by histology. Lesion volumes presented on the DWI MRI did not significantly differ from those on histology (1.53 ± 0.71 and 2.30 ± 0.13 ccm, respectively). Figure 4 illustrates the concordance between PCa on DWI MRI and histology and the coregistration of histology with the T_2_w MRI image.

**Figure 5:**
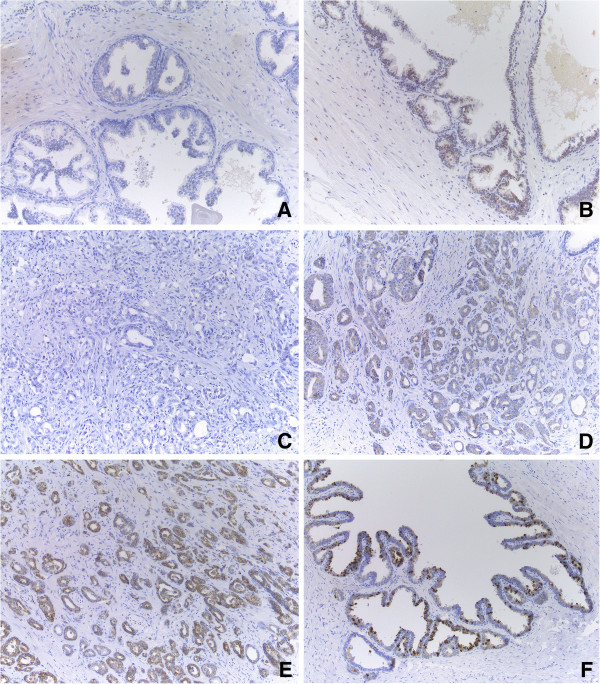
**Overview of CB1R expression in prostate tissue.** Prostate tissue sections were obtained after RP of four patients (original magnification ×100). In non-tumoural prostatic epithelium in general, no expression was seen **(A)**, although some randomly distributed areas of weak positivity were noted **(B)**. In PCa, the overall expression pattern was a mixture of negative **(C)** and weak positive **(D)** areas. Some foci of moderate positivity were randomly dispersed **(E)**. CB1R expression in the seminal vesicles was used as an internal positive control **(F)**.

In the specimen of the surgically removed prostate, the detection of CB1R expression as measured with IHC was comparable in the tissue sections of all four patients. The majority of the non-tumoural prostatic epithelium showed no CB1R expression (Figure 5A). Heterogeneously distributed throughout the tissue, some foci of weak expression were seen (Figure 5B), and very rarely, a few moderately positive glands were present. Strong CB1R expression was absent in the non-tumoural prostate parenchyma. CB1R was expressed in the cytoplasm of cells but not in nuclei and on the cell membrane.

In the PCa specimen, CB1R expression was overall absent or weak, though some areas of moderate CB1R expression were noted. Again, no/weak/moderate expression was heterogeneously distributed within the tumour (Figure 5C,D,E) and did not seem to be associated with the localization within the tumour or the prostate nor with the Gleason score. As in normal prostate cells, CB1R expression was observed in the cytoplasm while no positivity was seen in nuclei or cell membranes.

CB1R expression within the intraprostatic part of the seminal vesicles was used as an internal positive control (Figure 5F). As in all tissue sections, the epithelium of the seminal vesicles stained moderately to strongly for CB1R (cytoplasm).

### Metastatic PCa

**Figure 6:**
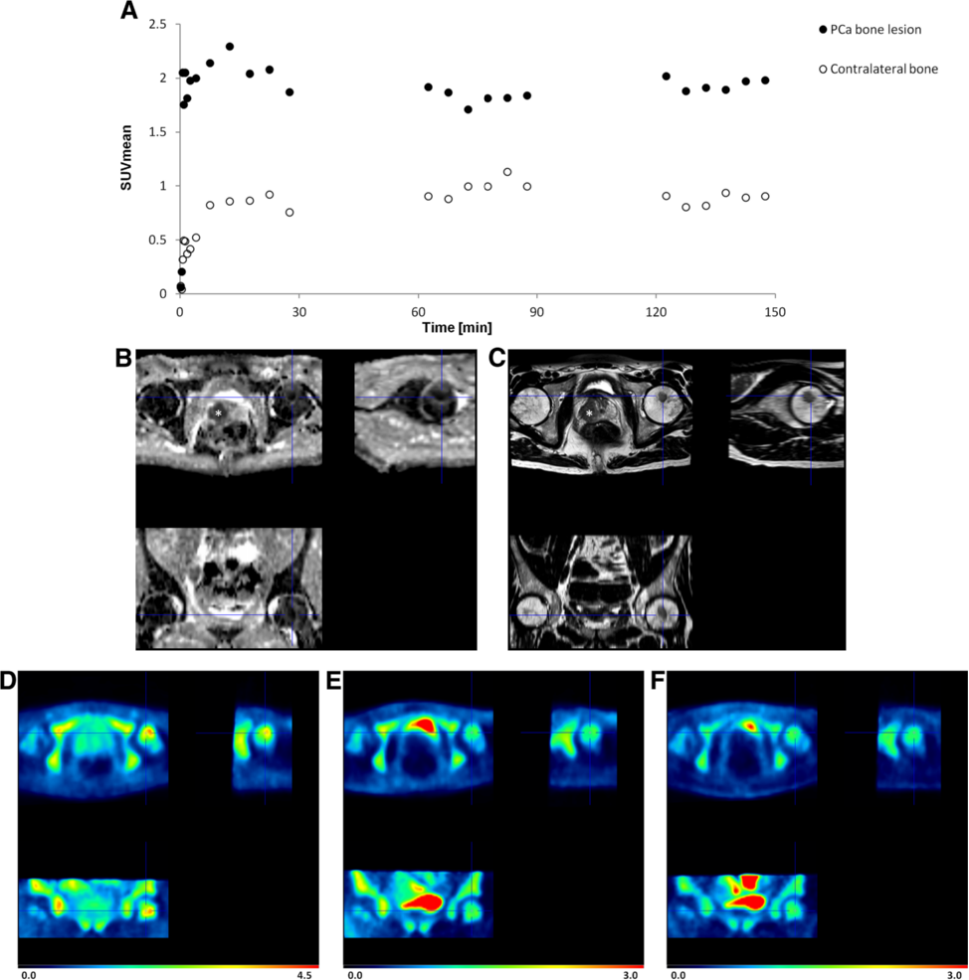
**Kinetics of **^**18**^**F-MK-9470 in a bone metastasis. **^18^F-MK-9470 uptake in a lesion of the left femoral head versus ^18^F-MK-9470 uptake in contralateral normal bone tissue of patient 5 **(A)**. The DWI MRI **(B)** and the T_2_w MRI **(C)** images show a low-signal-intensity mass in the lesion of the left femoral head as well as the local tumour (indicated with an asterisk). Integrated PET images of the first **(D)**, second **(E)** and third **(F)** acquisition time interval demonstrate increased ^18^F-MK-9470 uptake in the bone metastasis as compared to that of the contralateral bone. Note the absence of ^18^F-MK-9470 uptake in the local PCa visible on the MRI images. Data are scaled from 0 to 4.5 SUV in **(C)** and scaled form 0 to 3.0 SUV in **(D)** and **(E)**.

Increased uptake of ^18^F-MK-9470 over time was observed in a bone metastasis of a patient (patient 5) at initial diagnosis (Figure 6). Although the local tumour of this patient could not be differentiated from the surrounding benign tissue with ^18^F-MK-9470 PET, the metastatic lesion in the left femoral head was clearly visualized. The uptake of ^18^F-MK-9470 in the first, second and third acquisition time interval was 2.7, 1.8 and 2.2, respectively, times higher in the femoral lesion compared to that of the contralateral VOI.

**Figure 7:**
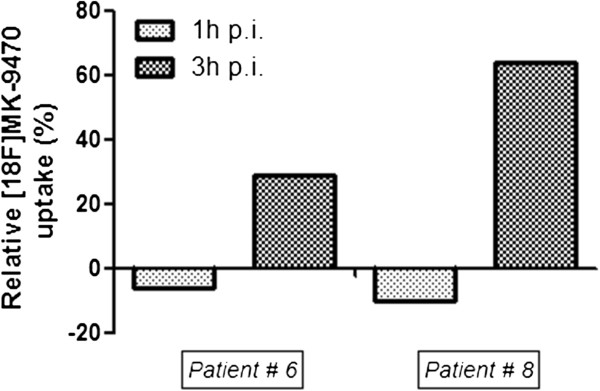
**Relative binding of **^**18**^**F-MK-9470 in lesions of the appendicular skeleton 1 and 3 h p.i.** Uptake of ^18^F-MK-9470 was increased with 29% (6) and 64% (8) in two metastases of the appendicular skeleton 3 h p.i.

**Figure 8:**
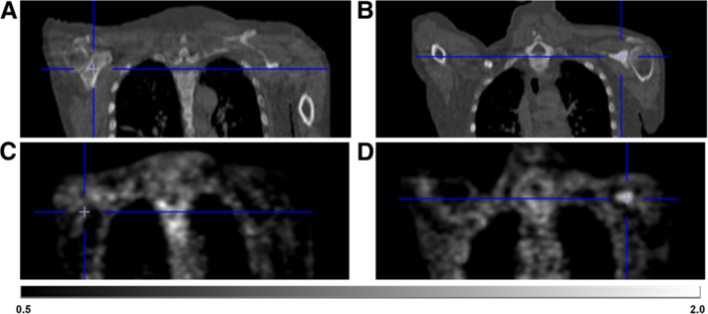
**Illustration of increased **^**18**^**F-MK-9470 binding 3 h p.i. in a metastatic lesion of the left scapula.** CT demonstrates normal bone of the right scapula **(A)** and bone metastasis in the left scapula **(B)**. As compared with the uptake in contralateral bone **(C)**, ^18^F-MK-9470 PET 3 h p.i. demonstrates increased uptake in the lesion of the left scapula **(D)**. Data are scaled from 0.5 to 2 SUV.

On the contrary, metastatic lesions in the axial skeleton showed lower ^18^F-MK-9470 uptake as compared with normal axial bone tissue. For example, ^18^F-MK-9470 uptake in tumoural versus normal bone was 1.77 ± 0.08 versus 3.12 ± 0.18 1 h p.i. (*p* < 0.01) and 1.69 ± 0.11 versus 2.28 ± 0.15 3 h p.i. (*p* < 0.05), respectively. However, compared to ^18^F-MK-9470 uptake in the contralateral bone tissue, tracer binding in a lesion of the left scapula (patient 8) and right humerus (patient 6) was 64% and 29%, respectively, which increased at 3 h p.i. (Figure 7). Figure 8 illustrates the higher intensity of ^18^F-MK-9470 binding 3 h p.i. in a metastatic lesion of the left scapula compared with the uptake in benign bone.

## Discussion

This study is the first clinical study to evaluate PET imaging of CB1R in an oncological setting. A PCa patient population with different disease stages was evaluated with the radioligand ^18^F-MK-9470 in order to evaluate the feasibility of CB1R imaging.

For locally confined primary PCa, ^18^F-MK-9470 PET could not differentiate malignant from normal prostatic tissue. A significantly higher ^18^F-MK-9470 uptake was observed in both prostate tissue compartments (benign and malignant) as compared to that of the muscle tissue. IHC findings did not clearly differentiate PCa from healthy prostate tissue based on CB1R expression patterns, which is in accordance with our PET findings. Though high CB1R expression has been shown in human PCa tissue cells [9,10], we could not confirm this finding in the current study. This may possibly be due to the small patient population. Moreover, in previous studies, CB1R expression has been validated at the subcellular level in human specimen obtained after surgery [9,10], whereas in this study, an *in vivo* molecular imaging technique was used. The measurement of CB1R mRNA levels and immunoreactivity are direct detection methods on a subcellular level. Nuclear molecular imaging of these structures could be hampered by problems reaching the intracellular target or a low expression level of the receptor despite an ubiquitously available mRNA. Also, CB1R was detected in the cytoplasm of prostate cells. To enable visualization of intracellular receptors, ^18^F-MK-9470 has to pass through the cell membrane. In view of its high lipophilicity and high uptake in brain [14], it is very likely that ^18^F-MK-9470 passes the cell membrane although this has not been demonstrated experimentally at the subcellular level. Malignant and benign lesion volumes delineated on the DWI MRI image were 1.53 ± 0.71 and 1.39 ± 0.58 ccm (mean ± SEM), respectively. Though a partial volume effect could be taken into account when detecting CB1R in both prostatic tissue compartments, we assume that in this study, the restricted CB1R density is mainly responsible for the inefficiency of ^18^F-MK-9470 PET.

The blood brain barrier prevents ^18^F-MK-9470 radiometabolites from entering the brain and thus reducing their non-specific binding. Since there exists no blood tissue barrier, addressing CB1R expression in PCa could suffer from a higher non-specific accumulation of these metabolites. Nevertheless, the two identified radiometabolites of ^18^F-MK-9470 showed negligible affinity for CB1R. Also, ^18^F-MK-9470 biodistribution in the five patients with primary PCa was comparable with previous findings [29] confirming normal tracer metabolism in these patients.

The second objective of this study was to investigate non-invasive detection of CB1R expression in PCa bone metastases. Whereas metastases in the axial skeleton could not be visualized due to high background uptake in the surrounding bone marrow, metastases in the appendicular skeleton showed higher ^18^F-MK-9470 uptake as compared to the uptake in their contralateral normal bone. In a previous study, CB1R expression seemed not to be associated with the Gleason score [9]. Therefore, one may assume that a considerably different expression level is presented on recurrent tumours as compared with that of the primary PCa, as well as among progressive bone metastases. Furthermore, compared to normal bone, a higher ^18^F-MK-9470 accumulation was observed in a metastasis early after tracer injection while increased uptake in distal tumours could only be observed at a late stage (3 h p.i.). Whereas this later observation could be due to defluorination and accumulation of free ^18^F-fluoride into osteoblastic bone metastases, no other areas with increased uptake in bone were observed in the current study or in previous biodistribution studies [20]. The reason for possible different uptake kinetics among metastases is currently not obvious. Quantification of ^18^F-MK-9470 uptake in bone can present additional challenges from that in the local prostate tissue due to the differences in blood flow. Further, several CB1R PET tracers have been developed for brain applications. Yet, to date, whole-body dosimetry in humans has only been validated for three tracers including ^18^F-MK-9470 [18,20]. Though features of suited tracers for peripheral imaging do not necessarily meet those for neurological imaging, the use of other developed CB1R tracers seems limited due to a low receptor affinity and/or high lipophilicity [13]. Despite none of the currently validated tracers is ideal for peripheral CB1R imaging, ^18^F-MK-9470 seems suited for imaging CB1R in PCa as previous reports demonstrated a high selectivity and affinity of ^18^F-MK-9470 for CB1R [20]. ^18^F-MK-9470 produces radiometabolites, which limit the specificity of the tracer for imaging CB1R in a peripheral setting [30]. Yet, both radiometabolites of ^18^F-MK-9470 have an extremely low affinity for CB1R. Thus, based on the abovementioned characteristics of ^18^F-MK-9470, we expect no unspecific binding of this tracer in bone metastases.

Since patients who met the inclusion criteria were rather rare and negative results were obtained in the first group of the total eight patients, it was decided to discontinue this study after the first data analysis. However, based on the current preliminary findings, ^18^F-MK-9470 PET could visualize peripheral bone metastasis. Still, this diagnostic technique will most likely not have an additional value as compared with the standard imaging techniques (standard bone scan and ^18^F-fluoride PET) currently used for detecting bone metastasis. Therefore, ^18^F-MK-9470 PET is probably not advantageous for the detection and evaluation of PCa. However, previous findings support the use of the ECS as a target for future treatment of CB1R-overexpressing tumours [11,12]. In view of selecting patients that could benefit from potential CB1R-targeted therapy approaches or investigating the efficacy of newly developed CB1R-based therapeutics *in vivo* in a (pre)clinical setting, imaging of CB1R with PET might help to select those patients benefitting from such a treatment.

## Conclusions

PET imaging with ^18^F-MK-9470 cannot detect local PCa and bone metastases in the axial skeleton but was able to visualize metastases in the appendicular skeleton. Future studies in larger patient populations need to clarify the heterogeneity regarding CB1R expression in PCa. However, from these pilot observations, it seems unlikely that ^18^F-MK-9470 PET will play a significant diagnostic role in PCa. There could be a potential role of ^18^F-MK-9470 PET for selecting patients who could benefit from CB1R-based therapies or for validating newly developed ECS-based treatments.

## Competing interest

The authors declare that they have no competing interests.

## Authors’ contributions

KME conceived of and contributed to the study design, acquired all data, performed the data analysis and interpretation and drafted the manuscript. FMM and CMD designed the study protocol. KVL and MK contributed substantially to the study design. MK and CC helped considerably with the data analysis. GMB performed the tracer labelling. KVL, CMD, FMM, MK, CC, GMB, KG and LM have been involved significantly in data interpretation, drafting the manuscript and revising it critically for important intellectual content. LVDB helped with the screening of patients and coregistration of the IHC data with the anatomical images. FC and LDW acquired the anatomical images and defined the prostatic tissue compartments in the images. EL coordinated and interpreted the histological and IHC examination of all resected prostate specimen. HVP, SJ, HD and KH helped with patient inclusion. LVDB, FC, LDW, EL, HVP, SJ, HD and KH contributed to the draft of the manuscript. All authors read and approved the final manuscript.
